# Serotonin-Induced Hypersensitivity via Inhibition of Catechol O-Methyltransferase Activity

**DOI:** 10.1186/1744-8069-8-25

**Published:** 2012-04-13

**Authors:** Douglas Tsao, Jeffrey S Wieskopf, Naim Rashid, Robert E Sorge, Rachel L Redler, Samantha K Segall, Jeffrey S Mogil, William Maixner, Nikolay V Dokholyan, Luda Diatchenko

**Affiliations:** 1 Department of Chemistry University of North Carolina, Chapel Hill, NC 27599, USA; 2 Center for Neurosensory Disorders, University of North Carolina, Chapel Hill, NC 27599, USA; 3 Department of Psychology, McGill University, 1205 Dr. Penfield Avenue, Montreal, QC, H3A 1B1, Canada; 4 Department of Biostatistics Gillings School of Global Public Health University of North Carolina, Chapel Hill, NC 27599, USA; 5 Department of Biochemistry and Biophysics, School of Medicine University of North Carolina, Chapel Hill, NC 27599, USA

## Abstract

The subcutaneous and systemic injection of serotonin reduces cutaneous and visceral pain thresholds and increases responses to noxious stimuli. Different subtypes of 5-hydroxytryptamine (5-HT) receptors are suggested to be associated with different types of pain responses. Here we show that serotonin also inhibits catechol O-methyltransferase (COMT), an enzyme that contributes to modultion the perception of pain, via non-competitive binding to the site bound by catechol substrates with a binding affinity comparable to the binding affinity of catechol itself (*K*_*i*_ = 44 μM). Using computational modeling, biochemical tests and cellular assays we show that serotonin actively competes with the methyl donor S-adenosyl-L-methionine (SAM) within the catalytic site. Binding of serotonin to the catalytic site inhibits the access of SAM, thus preventing methylation of COMT substrates. The results of in vivo animal studies show that serotonin-induced pain hypersensitivity in mice is reduced by either SAM pretreatment or by the combined administration of selective antagonists for β_2_- and β_3_-adrenergic receptors, which have been previously shown to mediate COMT-dependent pain signaling. Our results suggest that inhibition of COMT via serotonin binding contributes to pain hypersensitivity, providing additional strategies for the treatment of clinical pain conditions.

## Introduction

Catechol O-methyltransferase (COMT) is an enzyme that has been implicated in the perception of mechanical, thermal, and inflammatory pain in both humans and rodents [1-3]. Its association with a broad range of noxious stimuli suggests its critical role as an underlying factor for the fundamental processes of pain perception. The enzymatic activity of COMT is reversely correlated with the intensity of perceived pain; higher levels of COMT activity correspond to lower pain sensitivity [1-4]. Thus, compounds or cellular regulatory factors that lower COMT activity enhance pain sensation.

While commonly associated with antinociception [5,6], serotonin (5-hydroxytryptamine, or 5-HT) is also known to produce a hyperalgesic response when injected subcutaneously or into deep tissue [7,8]. Different subtypes of 5-HT receptors are associated with hypersensitive responses to various noxious stimuli. For example, mechanical hypersensitivity (allodynia) induced by serotonin has been linked to 5-HT1A, 5-HT2B, and 5-HT2C receptors [7,9], whereas the 5-HT2A receptor appears to be responsible for 5-HT-induced thermal hypersensitivity [10].

**Figure 1:**
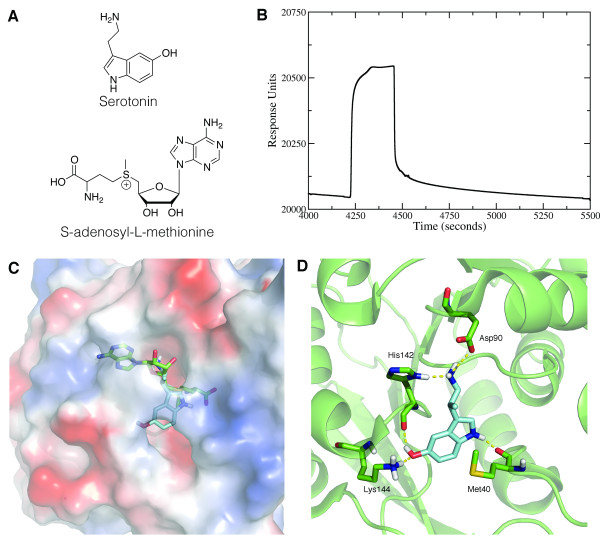
***In silico *and in vitro binding of serotonin and COMT.** ( **A**) Structures of serotonin and S-adenosyl-L-methionine (SAM). ( **B**) SPR data is fitted to association and dissociation curves to determine the kinetics of serotonin binding ( *k*_on_ = 0.0012 s^-1^, *k*_off_ = 13.7404 M^-1^ s^-1^). (**C** and **D**) Structural modeling of serotonin within COMT active site. ( **C**) Surface representation of serotonin binding to a pocket within COMT. Serotonin structure is shown in cyan; SAM structure is shown in green. The overlapped structures indicate that the amine sidechain of serotonin prevents SAM from actually binding to COMT. On the COMT surface, blue represents positively-charged, red represents negatively-charged, and white represents neutrally-charged regions. ( **D**) Interactions of serotonin with residues inside COMT active site. COMT structure and residues are shown in green; serotonin is shown in blue. Hydrogen-bonding interactions are highlighted with yellow-dashed lines.

A co-factor necessary for methylation of COMT substrates is S-adenosyl-L-methionine (SAM). SAM is responsible for donating the methyl group to a single hydroxyl group of the catechol substrate. Without SAM, COMT is unable to metabolize substrates such as norepinephrine and epinephrine, thereby leading to a potentiation of pain signaling through the downstream stimulation of β_2_ – and β_3_–adrenergic receptor pathways [2]. In comparing the chemical structures of SAM and serotonin (Figure 1A), we noted that the indole ring of serotonin is similar to the adenosine motif of SAM. Thus, we hypothesized that serotonin may play a role as a competitive inhibitor of SAM, which could provide a novel avenue for COMT-dependent pain modulation. Specifically, the inhibition of COMT activity through serotonin might reduce the ability of COMT to methylate endogenous catecholamine substrates, including several neurotransmitters, such as dopamine, epinephrine, and norepinephrine [11]. Here, we investigate the binding properties of serotonin to COMT using a combination of *in silico*, in vitro, and behavioral studies.

## Results

### Serotonin Binds to COMT

Binding interactions between serotonin and COMT were determined through surface plasmon resonance (Figure 1B) whereby both the rate of association (*k*_on_) and dissociation (*k*_off_) can be determined. We find that serotonin dissociates from COMT with a *k*_off_ rate of 0.0012 s^-1^, and binds to COMT with a *k*_on_ rate of 13.7404 M^-1^ s^-1^. The equilibrium dissociation constant (*K*_D_) was determined by dividing the dissociation rate from the association rate, and calculated to be 87 μM.

### Serotonin Computationally Docks to the COMT Active Site

Next, we investigated whether serotonin could be accommodated within the catalytic site of COMT. Using computational docking tools [12,13], we dock serotonin to an apo-COMT protein to predict its native pose in the active site. We find that serotonin occupies a site within the pocket that sterically overlaps with the binding site of SAM (Figure 1)C. In the crystal structure of holo-COMT, His142 participates in an edge-to-face interaction with the adenosine of SAM [14]. However, during serotonin binding, His142 donates a hydrogen bond with the amine side chain of serotonin (Figure 1D). Another hydrogen bonding interaction takes place with the serotonin amine and the side chain carboxyl group of Asp90. These two residues are responsible for positioning serotonin near the binding pocket of SAM, thereby preventing SAM from accessing its site. The carbonyl backbone of His142 also accepts a hydrogen bond from the hydroxyl group of serotonin.

Two more residues form hydrogen-bonding interactions with serotonin. The carbonyl of Met40 forms a hydrogen bond with the imidazole group of serotonin, while Lys144 forms a hydrogen bond with the hydroxyl group of serotonin. The side chain of Met40 also packs against the indole ring of serotonin. Overall, the results of this docking simulation suggest that serotonin may inhibit COMT activity by preventing the binding of the critical cofactor, SAM.

### Serotonin Inhibits COMT Activity

**Figure 2:**
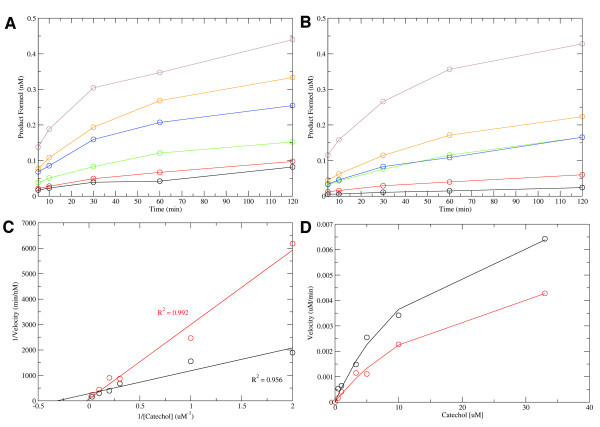
**Inhibition of COMT with serotonin.** ( **A**) and ( **B**) show the reaction rates of COMT at different catechol concentrations in the absence and presence of 100 μM serotonin, respectively. Legend: 0.5 μM (black), 1 μM (red), 3.3 μM (green), 5 μM (blue), 10 μM (orange), 33 μM catechol (brown). ( **C**) Lineweaver-Burk plot comparing the reciprocal velocities of activity against the reciprocal concentration of catechol in the absence (black) and presence (red) of 100 μM serotonin. The higher slope observed for serotonin indicates some level of inhibition. ( **D**) Michaelis-Menten determination of equilibrium inhibition constant. Activity assays in the absence (black) and presence (red) of 100 μM serotonin were fit to a two-site Michaelis-Menten model. While the *K*_M_ for catechol remains relatively the same, the *V*_max_ shows some noticeable differences thereby suggesting a non-competitive mechanism of inhibition between serotonin and catechol.

To test whether the binding of serotonin leads to inhibition of COMT activity, we performed a set of kinetics experiments to measure the reaction rates in either the presence or the absence of serotonin (Figure 2A,B). We measured the variance in reaction velocity with respect to changes in catechol concentration. A double-reciprocal plot comparing COMT velocities in the presence or absence of serotonin shows that addition of 100 μM of serotonin decreases the reaction velocity of COMT (Figure 2C). Thus, serotonin binding to COMT inhibits COMT activity.

We examined the kinetics of the observed inhibition in a set of experiments using a two-site Michaelis-Menten model (see Materials and Methods; Figure 2D). The Michaelis-Menten equilibrium constants for catechol binding were determined when serotonin was absent or present (100 μM) to provide the calculated values or the apparent *K*_*M*_ and *K*_M,app_ (see Materials and Methods). A comparison of the calculated *K*_M_ (16 μM) and *K*_M,app_ (18.5 μM) values shows a negligible difference between their affinities towards the catechol substrate. However, a comparison of the maximum velocities reveals a greater difference. The *V*_max_ in the absence of serotonin was 16 nM/min while the apparent *V*_max,app_ in the presence of serotonin was 7 nM/min, suggesting that serotonin inhibition is non-competitive with respect to catechol.

Given the differences between *V*_max_ and *V*_max,app_, we can calculate the inhibition constant for serotonin (apparent *K*_i_, or *K*_i,app_; see Materials and Methods). We find that *K*_i,app_ is 44 μM, which is within the vicinity of our approximate *K*_D_ determined via SPR (~87 μM). Overall, our results show that serotonin binds to COMT and inhibits its activity at a concentration that is similar to endogenous COMT substrates (~0.5 M intravesicular concentration; 25 μM reported for dopamine and ~10 μM for norepinephrine) [15-19].

### Serotonin-Induced Hypersensitivity is Diminished by Pretreatment with SAM or β_2/3_ Antagonists

If serotonin inhibition of COMT occurs through the competitive binding of the active site with SAM to produce pain hypersensitivity, we would predict that the administration of SAM should antagonize serotonin-induced hypersensitivity. We tested this hypothesis by performing a series of standard in vivo nociceptive assays in mice featuring the intraplantar (i.pl.) administration of serotonin [9]. We chose to use mechanical allodynia to assess the 5-HT-COMT interaction over the thermal pain, as thermal hyperalgesia is a far less important clinical symptom than mechanical allodynia, being observed in a much smaller percentage of chronic pain patients, being rated as much less bothersome, and having lower correlations with global pain ratings [20-22].

**Figure 3:**
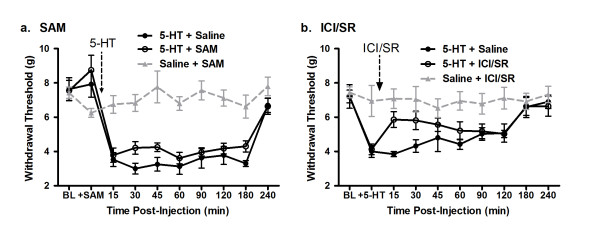
**Reduction of serotonin-induced hypersensitivity by SAM and β**_**2**_**/β**_**3**_**antagonism.** In all graphs, symbols represent mean ± SEM withdrawal threshold (g). In both experiments, serotonin (5-HT) was injected into one hind paw and withdrawal thresholds were assessed over a 4–h period. ( **A**) Pretreatment with 80 mg/kg SAM (5-HT + SAM) lowers 5-HT-induced hypersensitivity compared to saline-pretreated mice (5-HT + Saline). SAM alone (Saline + SAM) produced no changes in withdrawal threshold at any time point. ( **B**) Administration of the selective β_2_ and β_3_ antagonists, ICI118,551 and SR59230A (5-HT + ICI/SR), reduces 5-HT-induced hypersensitivity compared to saline controls (5-HT + Saline). ICI/SR alone (Saline + ICI/SR) produced no changes in withdrawal threshold at any time point.

Behavioral responsiveness to mechanical stimuli did not differ between groups prior to pharmacological manipulations, nor after SAM injection alone. I.pl. administration of serotonin (10 μM in a 20 μL injection volume) produced robust ipsilateral tactile hypersensitivity (i.e., mechanical allodynia) compared to saline (*p* < 0.001) (Figure 3A), consistent with previous observations [9]. No changes in mechanical withdrawal thresholds were observed in the contralateral hind paw (data not shown). Mice pretreated with SAM displayed modestly but significantly less serotonin-induced hypersensitivity compared to saline-pretreated mice (Figure 3A; *p* = 0.02).

As an additional approach to examine whether a COMT is associated with serotonin-induced hypersensitivity, we determined whether the blockade of downstream receptors known to mediate COMT–dependent changes diminishes serotonin-induced hypersensitivity. Since we have previously shown that low COMT activity leads to increased pain sensitivity via a β_2/3_-adrenergic mechanism, blocking β_2_ and β_3_ adrenergic receptors should reduce the serotonin-induced hypersensitivity if that sensitivity is COMT-mediated [2]. Again, serotonin produced robust hypersensitivity compared to saline-treated controls (*p* < 0.001; Figure 3B). Consistent with our hypothesis, we find that relative to serotonin administration alone, co-administration of ICI118,551 and SR59230A—compounds that respectively block β_2_ and β_3_ adrenergic receptors—significantly reduces serotonin hypersensitivity (*p* = 0.01; Figure 3B).

Thus, the results of our behavioral experiments are consistent with our in vitro and *in silico* results, and suggest that at least a portion of serotonin-induced hypersensitivity is mediated by a COMT → β_2/_β_3_ adrenergic receptor mechanism*.* Furthermore, our results indicate that serotonin can actively compete with SAM for the catalytic site of COMT.

## Discussion

### Biochemical Interactions of Serotonin and COMT

Using binding and activity assays, we show that serotonin interacts with COMT in a manner that inhibits enzymatic activity. We estimate that the COMT’s K_D_ for serotonin is approximately 87 μM, as determined with surface plasmon resonance experiments. Based on previous literature, our calculated *K*_D_ of 87 μM for serotonin is a reasonable [15-19]. SAM has the lowest *K*_D_ reported among COMT ligands, with a *K*_D_ of 20.2 μM [17]. In contrast, many common COMT catechol substrates exhibited dissociation constants in the range of 40 – 400 μM [17]. The higher dissociation constants observed for COMT substrates is most likely a consequence of its low substrate specificity. The binding site for catechol is very solvent accessible and thus can accommodate a large array of ligands [14]. Although COMT does not methylate most phenols or gallols [23], virtually all catechols are susceptible to methylation including bulkier steroid compounds. Thus, given a *K*_D_ for serotonin that is of the same order of magnitude observed for other known substrates, the interaction between serotonin and COMT is very plausible within cells that contain COMT.

We also find that the activity of COMT is reduced in the presence of serotonin, with a *K*_i_ of approximately 44 μM. Furthermore, our data suggest that serotonin is a non-competitive inhibitor with respect to catechol substrates. These results are in line with our structural model because serotonin occupies a region within the active site that appears to hinder SAM binding and still allows catechol binding from a steric viewpoint. Moreover, serotonin does not possess a methyl group to donate that would substitute for SAM binding.

### COMT Contributes to Serotonin-Induced Hypersensitivity

Our *in silico* models and in vitro results suggest that serotonin is an inhibitor of COMT. However, whether this inhibition has biological significance cannot be ascertained from these data alone. Our structural model suggests that serotonin inhibits COMT activity by actively competing with SAM at the active site. This mechanism is further supported by our kinetics studies. We performed behavioral experiments to determine if our *in silico* model and in vitro data are predictive of in vivo effects on pain behaviors. Mice pretreated with SAM displayed diminishes serotonin-induced mechanical hypersensitivity (Figure 3A). We hypothesize that the additional SAM produces this attenuation by decreasing the probability of serotonin occupying the active site of COMT. The relatively modest anti-allodynic effect is most likely due to the limited half-life of SAM [24]. The donating methyl group resides within a sulfonium center that is unstable due to its cationic charge. In addition, each SAM molecule can only donate one methyl group to a substrate. Once methylation occurs, SAM is converts into S-adenosyl-L-homocysteine (SAH). SAH can further serve as an inhibitor of COMT activity due to the high structural similarity to SAM but like serotonin, SAH is without a methyl donor [25].

SAM is a cofactor that is responsible for many methylation reactions within the cell. The behavioral effects of SAM found presently could be non-specific, and not mediated only by COMT. Thus, we examined whether serotonin enhances pain sensitivity via the downstream stimulation of a β_2/3_-adrenergic receptors as occurs in response to the pharmacological inhibition of COMT[2]. Inhibiting COMT, and thereby preventing methylation of epinephrine and norepinephrine, promotes pain signaling through the stimulation of β_2_ and β_3_ receptors [2]. If serotonin inhibits COMT in vivo, we would thus expect that the observed increases in pain sensitivity results, at least in part, from the activation of β_2/3_ receptors. Consistent with this view, blockade of β_2/3_ receptors inhibited serotonin-induced pain hypersensitivity in mice (Figure 3B).

### Implications for Serotonin-Induced Hypersensitivity and SSRIs

Our current results are of potentially substantial clinical significance. Increasing the bioavailability of serotonin with selective serotonin reuptake inhibitors (SSRIs) produces analgesia in some animal models [6,26] and can be used to treat clinical pain conditions [27,28]. However, SSRIs show relatively low analgesic efficacy in patients suffering from persistent pain conditions such as fibromyalgia, rheumatoid arthritis, and migraines [27][29-31] and have not gained widespread use for the treatment of persistent pain conditions [32-35]. In contrast, agents that have greater effects on the inhibition of norepinephrine relative serotonin reuptake (SNRIs), like duloxetine and milnacipran, show greater effect sizes and are more widely used for the treatment of a number of persistent pain states [36-38].

The clinical analgesic effects of SSRIs are very modest and do not match the expectations seen in animal models. Furthermore, several animal studies note a dual role for serotonin in both analgesia and hypersensitivity, which appears to be dependent on the model and site of administration. Thus, the unraveling of the neural mechanisms that underlie the dual action of serotonin on pain perception is of importance. Mechanisms proposed to date that explain the dual action of serotonin primarily involve the activation of different subpopulations of 5-HT receptors that are distributed at different anatomical locations [7-10]. Our *in silico,* in vitro and in vivo results suggest that serotonin’s pain-promoting effect can also at least in part be attributed to serotonin-dependent COMT inhibition. This finding opens a new avenue for increasing the analgesic efficacy of SSRIs by co-administrating SAMe and/or non-selective beta-blockers like propranolol.

## Conclusions

While several 5-HT receptors subtypes are known to contribute to pain perception, we have demonstrated that 5-HT action on COMT activity is another mechanism underlying 5-HT induced hypersensitivity, through a non-competitive binding process between SAM and serotonin at COMT’s catalytic site. Elucidation of the mechanisms of serotonin-induced hypersensitivity will contribute to the further understanding of its pharmacodynamics, and may lead to the future development of new and effective serotonin-based treatments for human pain conditions.

## Materials and methods

### Docking Simulations of Serotonin to COMT Active Site

We employ the MedusaDock package to generate possible ligand conformations within the protein active site, and utilize the MedusaScore package to evaluate each conformation generated by MedusaDock [12,13]. MedusaDock enables flexible docking of both the ligand and the side-chain amino acids of the protein. We perform all ligand docking simulations using the crystal structure of human COMT (PDB: 3BWM) [14].

Prior to all docking simulations, we first stripped all crystallized ligands bound to COMT. Crystallographic waters that were in the active site were retained for our docking simulations. We performed 200 docking simulations for serotonin, where each simulation began with a different seed number and each conformation generated from a simulation was subsequently minimized. All conformations were then ranked according to their free energy value. The lowest energy structure is determined to be the native pose.

### Binding Measurements using Surface Plasmon Resonance

Purified COMT protein (derived from porcine liver) and serotonin ligand were obtained from Sigma-Aldrich. Protein concentration was verified by performing a BCA assay (Thermo Scientific). Purified COMT was biotinylated at its surface lysine residues using EZ-Link NHS-LC-LC Biotin (Thermo Scientific) at a 1:1 mole ratio of biotinylating reagent to protein and subsequently purified using a 1 mL Sephadex G-25 medium spin column. Biotinylated COMT was buffered with 10 mM Tris pH 7.4, 1 mM MgCl_2_, 1 μM DTT and stored at 4°C until use.

Surface plasmon resonance (SPR) measurements were conducted at 25°C using a Biacore 2000 instrument with a previously established protocol [39]. Biotinylated COMT was first loaded onto a streptavidin-coated flow cell (sensor chip SA or Biotin CAPture Kit, GE Healthcare) at a flow rate of 5 μL/min, followed by a buffer flow, introduction of serotonin (buffered in 10 mM Tris pH 7.4, 1 mM MgCl_2_, 1 μM DTT), and then a final wash with buffer (10 mM Tris pH 7.4, 1 mM MgCl_2_, 1 μM DTT). Here the flow rate was established at 5 μL/min.

The dissociation rate of the serotonin-COMT complex was determined by fitting the dissociation data to a single-exponential decay.

(1) $$R=R_{o}\times e^{-k_{d}(t-t_{o})}$$

Here, the response unit from the SPR machine is denoted as *R*. Once the dissociation rate is determined, the association rate of serotonin to COMT can be found by fitting to the following equation:

(2) $$R=R_{\mathrm{eq}}[1-e^{-(k_{a}\left[\text{Serotonin}\right]+k_{a})(t-t_{a})}]$$

### Radiolabeled SAM Activity Assay

Activity of COMT was measured through a series of experiments where the enzyme was incubated with the substrate catechol (dissolved in H_2_O, Fisher Scientific) and S-[methyl-^3^ H]-adenosyl-L-methionine (PerkinElmer, 10.0 Ci/mmol) [40]. Reactions were carried out in a buffer containing 10 mM Tris (pH 7.4), 1 mM MgCl_2_, and 1 μM DTT with a total reaction volume of 100 μL.

To determine the linear range of the activity assay, SAM concentration (1 μM) was held constant while COMT concentration was varied (10 – 80 ng/μL). Each reaction was placed in a clean PCR tube and incubated at 37°C for 30 minutes. After incubation, reactions were terminated by addition of 100 μL of 1 M HCl to each tube. Radiolabeled catechol products were extracted from the reaction by adding 10 mL of scintillation fluid (MonoFlow I, National Diagnostics) and quantified using a scintillation counter. Activities were normalized for each reaction by performing a duplicate reaction with the COMT inhibitor, OR-486 (0.5 mg of inhibitor dissolved in 30 μL DMSO and 20 μL of 0.9% saline; OR-486 buffer alone does not inhibit reaction), and subtracting its radioactivity. We found that the linear range was between 10 and 40 ng/μL, and thus for all subsequent experiments we used a COMT concentration of 20 ng/μL.

Kinetic experiments were performed at a variety of catechol concentrations (0, 0.5, 1, 3.3, 5, 10, and 33 μM) while maintaining constant SAM (1 μM) and COMT (20 ng/μL) concentrations. Reactions were set-up for a variety of time points: 0, 5, 10, 30, 60, and 120 minutes. Each time-point reaction is incubated at 37°C and then quenched with 1 M HCl at its specified time point. Products were quantified as discussed above. Serotonin-inhibition experiments were conducted for the same catechol concentrations, but with the addition of 100 μM of serotonin (Sigma) to each reaction.

Kinetic parameters were determined by first fitting the data sets to a single-site Michaelis-Menten model (for reference), followed by a two-site Michaelis-Menten model as given by [17]:

(3) $$v=V_{\max}\frac{\left[\text{SAM}\right]\left[\text{Cat}\right]}{\left(\left[\text{SAM}\right]\left[\text{Cat}\right]+K_{m,\text{SAM}}\left[\text{Cat}\right]+K_{\text{m,Cat}}+K_{\text{D,SAM}}K_{\text{m,Cat}}\right)}$$

where catechol is abbreviated as “Cat.” To determine the mechanism of serotonin-inhibition, we calculated the apparent *K*_*i*_ (*K*_*i,app*_) of inhibition for both a competitive and non-competitive inhibitor, and compared which *K*_*i,app*_ yielded the lowest value. The apparent *K*_*i*_ for a competitive inhibitor is given by

(4) $$K_{\text{i,app}}=\frac{K_{\text{m,Cat}}\left[\text{Serotonin}\right]}{K_{\text{m,app}}-K_{\text{m,Cat}}}$$

where *K*_*m,app*_ is the apparent *K*_*m,*Cat_ for the serotonin-inhibited curve. The apparent *K*_*i*_ for a noncompetitive inhibitor is given by

(5) $$K_{\text{i,app}}=\frac{V_{\text{max,spp}\left[\text{Serotonin}\right]}}{V_{\max}}$$

where V_max,app_ is the apparent V_max_ for the serotonin-inhibited curve.

### Mouse Behavioral Experiments

Subjects were naïve, young adult (6–14 weeks old) outbred CD-1® (ICR:CrI) mice of both sexes (Charles River, Boucherville, QC). Animals were housed in groups of 2 to 6 with same-sex littermates, with food (Harlan Teklad 8604) and tap water available ad lib. Approximately equal numbers of male and female mice were used; no sex differences were noted so data from both sexes were pooled. All procedures followed the guidelines and regulations of the Canadian Council on Animal Care, and were approved by the McGill Downtown Animal Care and Use Committee.

Mechanical sensitivity was measured using the automated von Frey test. Mice were placed in individual transparent Plexiglas cubicles set atop a perforated metal floor (with 5-mm diameter holes 7 mm apart), and separated from each other by opaque dividers. Prior to testing, the mice were habituated to their surroundings for 1 h. A von Frey fiber with automatically increasing force (Ugo Basile Dynamic Plantar Aesthesiometer) was applied to the mid-plantar hind paw. Three separate withdrawal threshold determinations (on each hind paw) were taken at baseline (pre-injection) and then averaged. One withdrawal threshold determination (on each hind paw) was taken at every post-injection time point.

For the SAM pre-treatment study, after measuring baseline thresholds, mice received i.p. injections of S- (5’-Adenosyl)-L-methionine chloride (80 mg/kg) (Sigma), followed, 15 min later, by 20 μl i.pl. injections of serotonin hydrochloride (10 uM) (Sigma). Mice were tested at 15, 30, 45, 60, 90, 120, 180 and 240 min following the serotonin injection.

For the β-adrenergic antagonist study, mice received 20 μL i.pl. injections of serotonin hydrochloride (5 uM), followed, 15 min later, by i.p. injection of a cocktail of ICI118,551 (5 mg/kg) and SR59230A (50 mg/kg) (Tocris). Mice were tested at 15, 30, 45, 60, 90, 120, 180 and 240 min following the antagonist injection.

Pain hypersensitivity was quantified as the area over the time-threshold curve (post-injection), using the trapezoidal method.

### GEE Analysis

We utilize Generalized Estimating Equations (GEE) to determine whether SAM pretreatment or co-administration of SR and ICI treatment versus serotonin alone resulted in significantly different paw withdrawal thresholds [41] across the repeated measurements. The response was the repeated measurements of paw withdrawal threshold at fixed intervals post-administration of treatment. This model included an intercept and a binary covariate reflecting treatment status (e.g., ICI118,551 and SR59230A treatment versus serotonin alone) as the only covariate, where parameter estimates for this covariate can be interpreted as the overall mean change over measurements in PWT due to treatment. We assume an AR[1] correlation structure between repeated measurements, gaussian distribution of the data, and the identity link. We tested whether the estimate for our binary covariate for treatment was significantly different from zero (indicating a significant change in paw withdrawal threshold due to treatment effect) using Proc Genmod in SAS version 9.2, and report p-values from this test. We also report the estimated mean change in paw withdrawal threshold due to treatment effect ± the standard error of the mean change.

## Competing interests

The authors declare that they have no competing interests.

## Author’s contributions

DT performed the in silico simulations, SPR experiments, COMT activity assays, and wrote the paper. J.S.W. performed the mouse model studies and wrote the paper. N.R. performed the GEE analysis and wrote the paper. R.E.S. performed mouse model studies. R.L.R. performed SPR experiments. S.K.S. developed the COMT activity assay. J.S.M. designed the mouse model experiments and wrote the paper. W.M. conceived of the hypothesis, designed the activity assay experiments, and wrote the paper. N.V.D. designed the in silico experiments, developed the hypothesis, and wrote the paper. L.D. designed the activity assay experiments, developed the hypothesis, and wrote the paper.
